# Human Disturbance during Early Life Impairs Nestling Growth in Birds Inhabiting a Nature Recreation Area

**DOI:** 10.1371/journal.pone.0166748

**Published:** 2016-11-16

**Authors:** Carolina Remacha, Juan Antonio Delgado, Mateja Bulaic, Javier Pérez-Tris

**Affiliations:** 1 Department of Zoology and Physical Anthropology, Complutense University of Madrid, Madrid, Spain; 2 Department of Ecology, Complutense University of Madrid, Madrid, Spain; Universita degli Studi di Milano-Bicocca, ITALY

## Abstract

Nature recreation conflicts with conservation, but its impacts on wildlife are not fully understood. Where recreation is not regulated, visitors to natural areas may gather in large numbers on weekends and holidays. This may increase variance in fitness in wild populations, if individuals whose critical life cycle stages coincide with periods of high human disturbance are at a disadvantage. We studied nestling development of blue tits (*Cyanistes caeruleus*) in a natural area where recreation activities intensify during weekends and other public holidays at picnic and leisure facilities, but not in the surrounding woods. In nests located near recreation facilities, blue tit nestlings that hatched during holidays developed slowly, and fledged with low body mass and poor body condition. However, nestlings that hatched outside of holidays and weekends in these nest boxes developed normally, eventually attaining similar phenotypes as those hatching in the surrounding woods. Within-brood variance in body mass was also higher in broods that began growing during holidays in disturbed areas. Our results show that early disturbance events may have negative consequences for wild birds if they overlap with critical stages of development, unveiling otherwise cryptic impacts of human activities. These new findings may help managers better regulate nature recreation.

## Introduction

Nature recreation is becoming increasingly popular, and leisure facilities have proliferated in natural areas as a consequence [1]. Recreational activities may impact wildlife if human disturbance alters animal adaptive behaviours [2–3], or reduces accessibility to breeding or foraging habitats [4]. These impacts may prevent the settlement of animals in recreation areas, or may reduce their survival or breeding success [5–9].

Understanding the impacts of recreation on wildlife is crucial if management of natural areas aims to reconcile conservation and visitor expectations. However, recreation impacts may intricately vary in both space and time. Most visibly, visitors usually gather during public holidays (weekends and other non-working days) in areas with unrestricted public access [10]. Such fluctuations are known to affect wildlife. For instance, during crowded holidays, some species avoid using the areas that are most frequented by people [11–13]. The impact of disturbance may be greatest on breeding individuals and their offspring, which are behaviourally constrained by the location of their breeding sites and parental care demands. However, whether temporal exposure to human influence during holidays impairs breeding performance of wildlife has yet to be investigated.

Intense recreation activity during holidays may repeatedly affect wild populations during the breeding season, but the importance of its impact on individual fitness will probably depend on the stages of the life cycle that are affected [14]. For example, disturbance during early development may have negative effects later in life [15], as has been particularly well documented for altricial birds. In these species, nestling growth and subsequent survival greatly depend on parents’ dedicated brooding and provisioning of hatchlings, whose metabolic efficiency is constrained by ectothermy [16–17]. Therefore, temporal fluctuations in the impact of recreation might increase variance in fitness in wild populations, putting individuals whose critical stages of development overlap with periods of high human disturbance at a disadvantage.

We studied nestling growth of a small passerine, the blue tit (*Cyanistes caeruleus*), in a natural area where recreation is not regulated. This results in differences between holidays and working days in the number and extent of visitors. The blue tit is a model species in studies of avian breeding ecology and life-history evolution, aiding the interpretation of variation in breeding performance in relation to the spatiotemporal distribution of recreational activities. In addition, blue tits are fairly common in nature recreation areas and other humanised environments in Europe, making the species a good model for the analysis and monitoring of recreation impacts on wild populations. In our study area, human impact varies between areas of the forest equipped with recreation facilities (where visitors gather on holidays) and the surrounding woods (where disturbance is limited to hikers passing by nest locations). We set out to test whether blue tits develop more poorly if they begin growth under conditions of increased human disturbance during holidays. To this end, we compared nestling growth and survival of blue tits that initiated post-hatch development on holidays or on non-holiday weekdays (hereafter, working days) in nest boxes subjected to recreation disturbance (those located near recreation facilities) or free of this source of stress (quiet nest box locations in the surrounding woods).

## Methods

### Ethics Statement

Our field methods adhere to the ASAB/ABS Guidelines for the treatment of animals in behavioural research and teaching (doi: 10.1016/j.anbehav.2011.10.031), and were approved by the Area for Flora and Fauna Conservation of the Regional Government of Madrid (permit number 530408/2009) and the Spanish National Heritage Authority.

### Study area and field methods

We studied blue tits in an oak forest (*Quercus pyrenaica*) located 60 km from the city of Madrid, central Spain (La Herrería, 40° 34’ N, 4° 9’ W). The forest is a very popular recreation area managed by Spanish National Heritage, and includes a 7.5-ha picnic zone cleared of understory, set with wooden tables and benches and crossed by a small paved pathway allowing road traffic. Public use of this area follows the typical pattern in nature recreation facilities where visitor numbers are not regulated. During spring, large numbers of visitors gather there during weekends and other non-working days. During such days, up to 700 people use the recreation facilities from early morning to sunset, with groups of people often settling on fix spots, where they practice leisure activities that may disturb the birds nesting nearby. Road traffic and biking also increase heavily during holidays. However, few visitors go into the surrounding woods, where they only mildly affect breeding blue tits by walking past their nests along hiking trails. During working days, the forest is often visited by organized groups of school children, who use the picnic area mainly during midday lunch.

We classified nest boxes as disturbed (nest boxes located near recreation facilities, where visitors congregate and permanently disturb nests during holidays) or quiet (those located in the woods, where human presence near boxes is rare). Therefore, quiet nest boxes act as a control in our study, representing nearly natural conditions of low human impact. Importantly, the heterogeneous regime of visits to the forest, with clear-cut differences between holidays and working days in areas set for recreation, provides an excellent opportunity to test for human impacts on nestling growth. Because the conditions in which nestlings initiate post-hatching development critically determine subsequent growth [15], we classified blue tit broods into two groups: holiday broods (those whose first two days post-hatching overlapped with weekends or other non-working days), and working-day broods (the rest, which initiated growth in periods of low visitor affluence). Thus, if human disturbance impairs blue tit growth, we expected to observe impaired growth only in holiday broods hatched in disturbed nests. However, working-day broods hatched in disturbed nests should escape the effects of intense disturbance during periods of visitor crowding. Therefore, the latter should experience similar growing conditions as those of broods hatched in quiet nests on any day.

**Fig 1:**
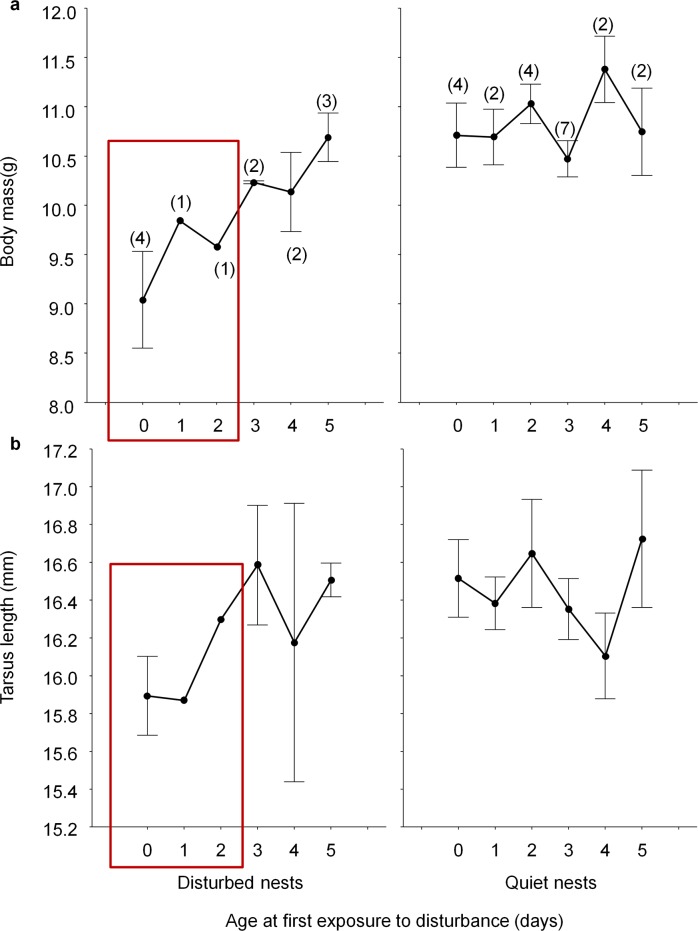
Variation in fledgling traits in relation to age at first exposure to disturbance. Fledgling body mass (a) and tarsus length (b; mean ± se) of nestlings with different ages at first holiday. Sample sizes are shown in brackets. The red boxes include the time window considered to represent early development in our analyses (two-post hatching days).

We used two-posthatching days as the time window to consider nestlings early disturbed. Although the initial idea was to use broods hatched during holidays as the only holiday broods, final sample size for some groups of nest boxes turned out to be too small, and therefore we decided to consider up to two days post hatching as a reasonable time window for early growth. In fact, this classification is a priori against our statistical power, because the expected effects should be more difficult to detect if the impact of human disturbance decreases with age at first exposure, given that nestlings that are first disturbed at age two days are closer to non-exposed nestlings than those disturbed as hatchling (Fig 1).

During spring 2009, we monitored 65 disturbed nest boxes (which were set for this study during the previous winter) and 73 quiet nest boxes (which had been set a few years before the study to monitor forest bird populations; these nest boxes were deliberately placed in quiet locations away from recreation facilities precisely to avoid human disturbance). The number of nest boxes was determined based on previous experience of nest box occupation by blue tits in the forest, while at the same time attempting to avoid unrealistically increasing the availability of holes suitable for nesting (minimum distance between neighbour nest boxes ≈ 20 m; our study area is a mature forest where the availability of natural nest holes exceeds that of nest boxes; C. Remacha, pers. obs.). The number of nest boxes that may thus fit in the forest (especially around recreational facilities) necessarily limited the sample size available for this study. Nevertheless, given the amount of nest manipulation necessary for the study (a minimum of nine visits to each nest, including measuring nestlings in five occasions starting at age 2 days), we also wanted to keep sample size to the minimum necessary to detect biologically relevant effects. Our reasons were both ethical (to reduce the number of nests subjected to invasive procedures) and practical: the same person (CR) measured all nestlings, thereby avoiding biasing intra- vs inter-brood comparisons, as would occur if different observers shared nest-box monitoring workload (which would have required greater sample size to maintain statistical power). Assuming an average nestling mass of 10.4 g (S.D. = 0.81) for the population, the sample size necessary to detect a 10% deviation from the population mean with α = 0.05 (two-tailed) and initial power = 0.90 was *n* = 9 birds per group, which gives *n* ≈ 36 nest boxes as a reasonable sample size for our 4-group comparison. It is important to note that disturbed and quiet nest boxes did not represent different study sites but were located in the same forest. However, the location of recreational facilities northeast of the forest led to spatial clumping of disturbed nests, although many of these were more closely located to quiet nests than to other disturbed nests, and *vice versa*. More importantly, holiday and working-day broods showed no spatial patterning of any kind (S1 Fig).

Starting in early April, we regularly checked nest boxes to find active blue tit nests. Size and completion date of all clutches were determined by visiting nests repeatedly during the laying period. We also recorded laying date (the day the first egg was laid, assuming that one egg was laid per day) and hatching date (the day the first hatchling was hatched) of each clutch. We determined exact hatching dates by visiting nests one day before expected hatching (14 days after the penultimate egg was laid), and then every day until hatching occurred. When one of the two days post-hatching was a Saturday, a Sunday or any other public holiday, the brood was classified as a holiday brood as opposed to a working-day brood.

Hatchlings were individually marked at age 2 days (hatching day = 0) by colouring the ventral sides of their wings with distinct colour codes, using harmless felt-tip markers. At age 8 days, we marked nestlings with standard aluminium rings. We weighed nestlings (to the nearest 0.01 g) at ages 2, 5, 8, 11 and 15 days. At ages 8, 11 and 15 days we also measured nestling tarsus length (to the nearest 0.01 mm). At age 15 days (when they were considered as fledged) we took blood samples to determine nestling sex using molecular tools [18].

In tits, nestling mass prior to fledging is positively correlated with offspring survival [19–20]. Therefore, we analysed in further detail the condition of nestlings at age 15 days. We regressed body mass on tarsus length (*r* = 0.53, F_1,189_ = 73.30, *P* < 0.001) and used the residuals as an index of fledgling body condition [21]. When two or more nestlings survived to age 15 days within a brood, we computed the coefficient of variation (CV) of tarsus length and body mass to measure sibling inequalities, which were interpreted as additional evidence of unfavourable rearing conditions [22].

In order to control for possible effects of parental quality on nestling development, we captured both parents with flap traps set inside nest boxes when they were feeding 7–9 day old nestlings. Captured parents were sexed (females had brood patches but males did not) and aged by plumage as first breeders or older birds [23]. They were measured (tarsus length and body mass, with the same accuracy as for nestlings) and marked with aluminium rings before being released near the nest. To further control for possible confounding effects, we also recorded average temperature and rainfall during the two days after hatching of each brood, using data from a meteorological station located within the study area.

### Statistical analyses

If recreation affects nestling development, we expect to find an interaction between the effects of type of nest (disturbed or quiet) and type of brood (holiday or working-day), as holiday broods from disturbed nests are predicted to be the only ones showing impaired development in our study area.

Meteorological conditions and breeding time may influence breeding performance in birds [24]. Therefore, to avoid the confounding effects of these temporal factors on the effects of human disturbance, we tested for variation in hatching date, temperature and rainfall (measured during the two days post-hatching) between nest types and brood types. We used ANOVA to analyse variation in hatching date and temperature. The scarce rainfall events during the study period better fitted to a binomial distribution (rainfall occurrence), and were analysed using generalized linear models with binomial error and logit link function (results shows in S1 Material).

We also analysed variation between nest types and brood types in clutch size (as a measure of the initial parental investment) and parental traits (separately for each sex). Variation in clutch size, body mass and tarsus length was analysed using ANOVA. Possible non-random distribution of first breeders and older birds among nest types or brood types was tested using log-linear analyses (results shows as S1 Material).

The effect of holiday disturbance on nestling development (body mass and tarsus length) was tested using linear mixed models. We included the identity of each nestling nested within brood identity as random factors, thus taking into account intra-individual variation in nestling traits during the nesting period and the non-independence of individuals from the same brood. Nestlings that died before age 15 days were excluded from the analysis. Each model included the following fixed effects: brood type (holiday or working-day), type of nest (disturbed or quiet), nestling age (in days), sex and all their interactions. In this analysis, the three-way interaction between nestling age, brood type and type of nest tested our prediction that variation in nestling growth trajectories associated with holiday disturbance had a different magnitude in disturbed (where an effect is expected) and quiet nest boxes (where no effect is expected). When such interactions were detected, we tested pairwise differences between groups.

To assess which type of curve best fit to our body mass growth data we used the package FlexParamCurve version 1.5–3 [25]. We performed linear mixed models with the main curve parameters (“A” as the asymptote of the increasing curve, “k” as the rate of change of growth rate with age and “Infl” as the age when nestlings growth at maximum rate).

At age 15, we performed linear mixed models to test for effects of brood type and type of nest on fledgling body mass, tarsus length and body condition, and ANOVA to test for differences in within-brood variation in fledgling size and mass. Nest success (whether or not the brood produced at least one fledgling) was analysed using generalized linear models, and individual nestling survival was analysed using linear mixed models, in both cases with binomial error and logit link function.

Mixed models were assessed using the package lme4 [26]. In all analyses we used nestlings as sample units, and nest identity was included as a random factor. We included laying date or brood size as covariates when they significantly contributed to the model. We tested both linear and quadratic effects of laying date [27], which was log-transformed in order to improve normality of the residuals (the use of the untransformed variable produced an excess of negative residuals, arguably because phenological timing was not linearly related to calendar dates). Non-significant interactions or covariates were excluded from models through a stepwise elimination procedure (lmerTest package) [28]. We tested significance of the effects in the final model by log-likelihood ratio test (LRT) comparison between nested models (saturated model versus reduced model). When an interaction was significant, we used Tukey post-hoc test (lsmeans package) [29] to test for pairwise differences between levels of the relevant factors. All statistical procedures were performed using R (version 3.3.0).

## Results

Fifty-five nest boxes occupied by blue tits were monitored, of which 21 were disturbed nests and 34 were quiet nests (S2 Fig). Only one disturbed nest failed before hatching.

### Breeding success

Out of 366 hatchlings, 195 fledged (from 36 nest boxes). Nest success decreased through the season (effect of log-laying date: χ^2^_(1)_ = 8.05, *P* < 0.01), but did not vary between nest types (χ^2^_(1)_ = 1.31, *P* = 0.25) or brood types (χ^2^_(1)_ = 0.61, *P* = 0.44; interaction: χ^2^_(1)_ = 2.43, *P* = 0.12). Nestling survival to age 15 days decreased also through the season (effect of log-laying date χ^2^_(1)_ = 30.41, *P* <0.001, estimate ± se = -19.09 ± 5.48), and was higher in disturbed than in quiet nests (χ^2^_(1)_ = 4.26, *P* = 0.04, estimate ± se = -3.65 ± 1.85, level quiet nest). There were no differences between holiday and working-day broods (χ^2^_(1)_ = 0.19, *P* = 0.66, brood type × nest type: χ^2^_(1)_ = 3.29, *P* = 0.07).

### Nestling growth and fledgling condition

#### Nestling growth

**Fig 2:**
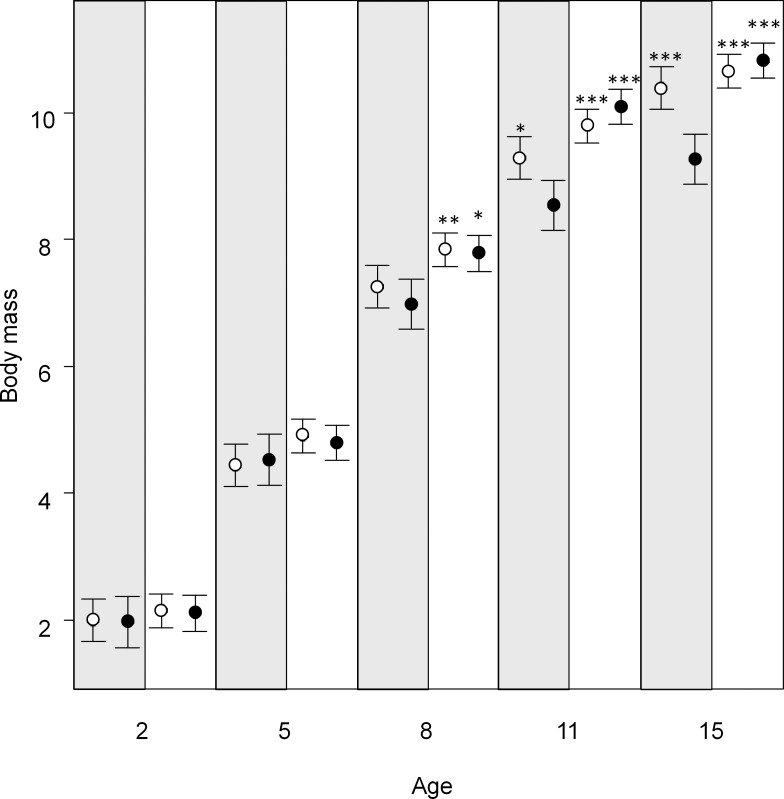
Body mass of blue tit nestlings measured at different ages in quiet and disturbed nest locations. Marginal means of body mass ± confidence intervals at each age obtained from linear mixed models. Filled circles represent holiday broods, while open circles represent working-day broods. Quiet nest boxes have a grey background. Stars indicate statistical significance of post-hoc comparisons of the corresponding mean with the mean of disturbed nests-holiday broods (*P < 0.05, **P < 0.01, ***P < 0.001). Working-day broods in disturbed nests did not differ from broods in quiet nests (whether holiday or working-day; all comparisons had a *P* value > 0.10).

We monitored growth between ages 2 and 15 days of 191 nestlings (90 males and 101 females from 34 nest boxes). Significant three-way interactions between type of nest, brood type and nestling age (controlling for the effects of laying date and brood size; Table 1) showed that nestlings in disturbed nests developed more slowly if they initiated growth on holidays. Working-day broods hatched in disturbed nests showed normal development, as they attained growing trajectories similar to broods from quiet nests (whether holiday or working-day; Fig 2). Delayed growth of holiday broods in disturbed nests could be detected at age 11 days for body mass (Post-hoc Tukey test contrast with working-day broods in disturbed nests df = 36.7; t = 2.95 *P* = 0.03, with holiday broods in quiet nests df = 36.22; t = 6.31; *P* < 0.001, and with working-day broods in quiet nests df = 36.9; t = 5.50; *P* < 0.001).

**Table 1:** Likelihood ratio tests for the effects of brood type and nest type on nestling traits measured at different ages after removal of not significant terms (reduced models).

	Body mass		Tarsus length	
	χ^2^	*P*	χ^2^	*P*
**Between-subject effects:**				
Type of brood	(1) 1.77	0.18	(1) 0.95	0.33
Type of nest box	(1) 16.45	<0.001	(1) 3.09	0.08
Sex	(1) 20.63	<0.001	(1) 29.47	<0.001
Type of brood × Type of nest	(1) 2.54	0.11	(1) 1.62	0.20
(Log-Laying date)^2^	(1) 8.09	0.004	(1) 8.13	0.004
Log-Laying date			(1) 11.00	<0.001
Brood size			(1) 2.56	0.11
**Within-subject effects:**				
Age	(4) 2772.6	<0.001	(2) 617.68	<0.001
Type of brood × Age	(4) 34.91	<0.001	(2) 21.85	<0.001
Type of nest × Age	(4) 125.22	<0.001	(2) 3.71	0.16
Sex × Age	(4) 64.44	<0.001		
Type of brood × Type of nest × Age	(4) 96.15	<0.001	(2) 20.55	<0.001
(Log-Laying date)^2^ × Age	(4) 51.04	<0.001	(2) 10.67	0.005
Log-Laying date × Age			(2) 13.53	0.001
Brood size × Age			(2) 9.02	0.01

Repeated measures (within-nestling effects estimated at different nestling ages) mixed-models of variation in body mass and tarsus length in relation to type of nest (disturbed or quiet), type of brood (holidays or working-day), and nestling sex. Laying date (linear or quadratic effects) and brood size were included as covariates when significant, and brood identity was a random factor (effect not shown). Chi-square statistics are preceded by their respective df (in brackets).

#### Growth curve parameters

The growth curve that best fitted our data was logistic. Holiday broods hatched in disturbed nests attained lower asymptotic mass (A parameter, marginal mean ± se; 9.69 ± 0.24 g) than other groups (working day broods-disturbed nests 10.84 ± 0.22 g, df = 29.9, t = 3.48; *P* = 0.008, holiday broods-quiet nests 11.16 ± 0.19 g, df = 29.71; t = 4.81; *P* < 0.001 and working day broods-quiet nest 11.00 ± 0.18 g, df = 29.7; t = 4.37; *P* < 0.001, Table 2).

**Table 2:** Stepwise model selection for parameters of the body mass growth curve.

	A	Infl	k
Type of brood	χ^2^ = 11.69; *P* < 0.001	χ^2^ = 8.06; *P* = 0.004	χ^2^ = 0.10; *P* = 0.75
Type of nest box	χ^2^ = 0.37; *P* = 0.54	χ^2^ = 3.27; *P* = 0.07	χ^2^ = 0.47; *P* = 0.49
Sex	χ^2^ = 58.51; *P* < 0.001	χ^2^ = 2.57; *P* = 0.11	χ^2^ = 0.32; *P* = 0.57
Type of brood × Type of nest	χ^2^ = 9.69; *P* = 0.002	χ^2^ = 7.79; *P* = 0.005	Removed
Type of nest × Sex	Removed	Removed	Removed
Type of brood × Sex	Removed	Removed	Removed
Type of brood × Type of nest × Sex	Removed	Removed	Removed
(Log-Laying date)^2^	Removed	Removed	Removed
Log-Laying date	Removed	Removed	Removed
Brood size	Removed	χ^2^ = 4.66; *P* = 0.03	Removed

All likelihood ratio tests of fixed effects with df = 1. A is asymptotic mass, k is the rate of change of growth rate with age and Infl is the inflection point of the growth curve, which represents age at maximum growth rate.

At the same time, age at maximum growth rate was earlier in these broods (Infl parameter: 5.24 ± 0.22 days) than in broods hatched in disturbed nests on working days (6.08 ± 0.20 days; df = 28.5; t = 2.78; *P* = 0.04). However, there were no significant differences with broods from quiet nests hatched on working days (5.63 ± 0.17 days, df = 28.7; t = 1.43; *P* = 0.49) or holidays (5.84 ± 0.17 days, df = 28.4; t = 2.15; *P* = 0.16, Table 2).

The rate of change of growth rate with age (k parameter) was similar between broods (Type of brood × Type of nest, F_1,29.1_ = 0.05, *P* = 0.83, Table 2).

#### Fledglings traits

For body mass, the three-way interaction “Type of brood × Type of nest box × Sex” was significant (Table 3). Both female and male nestlings from disturbed nests fledged with lower body mass than all other nestlings in the study (all post-hoc Tukey tests with *P* < 0.03). Differences between groups were larger in females than in males (S3 Fig)

**Table 3:** Stepwise model selection for the effects of disturbance on fledgling traits (LRT results).

	Body mass	Tarsus length	Body condition
Type of brood	χ^2^ = 8.96; *P* = 0.003	χ^2^ = 4.07; *P* = 0.04	χ^2^ = 7.51; *P* = 0.006
Type of nest box	χ^2^ = 1.27; *P* = 0.26	χ^2^ = 0.05; *P* = 0.82	χ^2^ = 2.42; *P* = 0.12
Sex	χ^2^ = 12.45; *P* < 0.001	χ^2^ = 61.34; *P* < 0.001	χ^2^ = 5.43; *P* = 0.02
Type of brood × Type of nest box	χ^2^ = 7.36; *P* = 0.007	χ^2^ = 6.17; *P* = 0.01	χ^2^ = 5.54; *P* = 0.02
Type of nest box × Sex	χ^2^ = 0.0008; *P* = 0.98	Removed	Removed
Type of brood × Sex	χ^2^ = 3.53; *P* = 0.06	χ^2^ = 4.50; *P* = 0.03	Removed
Type of brood × Type of nest box × Sex	χ^2^ = 4.32; *P* = 0.04	Removed	Removed
Log-Laying date	χ^2^ = 4.69; *P* = 0.03	χ^2^ = 17.07; *P* < 0.001	Removed
(Log-Laying date)^2^	Removed	χ^2^ = 13.20; *P* < 0.001	Removed
Brood size	Removed	χ^2^ = 7.42; *P* = 0.006	χ^2^ = 6.06; *P* = 0.01

Linear mixed models of variation in nestling body mass, tarsus length and body condition at age 15 days, in relation to type of nest (disturbed or quiet), type of brood (holidays or working-day), and nestling sex. Laying date and brood size were included as covariates, and brood identity was a random factor (effect not shown). Effect degrees of freedom equalled 1 in all cases.

Slow-growing holiday nestlings from disturbed nests fledged with worse body condition than nestlings from quiet nests (post-hoc Tukey test with *P* < 0.001; Table 3). The same tendency was found when the former were compared with nestlings from working-days broods in disturbed nests, but this difference was marginally not significant (df = 28.92; t = 2.65; *P* = 0.06; Table 3). Tarsus length of fledglings only differed between holiday broods in disturbed nests and holiday broods from quiet nests (post-hoc Tukey test df = 24.50; t = 2.86; *P* = 0.04, all other comparison with *P* > 0.18). There were not significant differences in tarsus length between holiday-working broods within each sex (all post-hoc Tukey test with *P* > 0.3).

#### Sibling inequalities

The analysis of within-brood CV of body mass produced a significant interaction between nest type and brood type (F_1,28_ = 8.31; *P* < 0.01), which revealed unfavourable rearing conditions for holiday broods in disturbed nests. Sibling inequalities in relation to body mass were larger for holiday broods compared to working-day broods in disturbed nests (post-hoc test; t = 3.22; *P =* 0.02), and also compared to broods of either type from quiet nests (post-hoc tests: all *P* values < 0.005). Holiday and working-day broods showed similar CV of body mass in quiet nests (post-hoc test, t = 0.56; *P* = 0.94). The within-brood CV of tarsus length did not vary in relation to human disturbance (effect of nest type: F_1,28_ = 0.02, *P* = 0.88; brood type: F_1,28_ = 3.81, *P* = 0.06; interaction: F_1,28_ 0.02; *P* = 0.89).

## Discussion

Our study shows that short periods of intense human disturbance during early life may impair nestling development in the blue tit. We found various lines of evidence in support of this idea. Blue tits that hatched in disturbed nests had a worse start in life if they initiated post-hatching development on holidays, when visitors gather in picnic areas and exert a constant influence on disturbed nests. The affected nestlings developed slowly (as shown by low body mass from age 11 days onwards) and fledged in worse body condition, also demonstrating greater sibling inequalities. However, blue tits that started growing on working days in disturbed nests avoided disturbance impacts, and fledged with similar characteristics as those that hatched in the quiet nest boxes where human influence was always low.

Impaired growth of nestlings that were heavily disturbed early in their lives may be the consequence of adult antipredatory behaviours conflicting with parental care [30] or direct stress suffered by nestlings [31]. Faced with increased predation risk associated with the presence of humans, birds may favour their own survival at the expense of their offspring’s fitness [32–33]. Antipredatory behaviours to humans, such as churring calls and hesitation to enter the nest box, have been reported in blue tits ([34], C. Remacha, pers. obs.) and may thus decrease nest attentiveness, a response that may have its greatest impact during critical periods of nestling development. In our study, breeding pairs affected by sustained human disturbance during holidays may have decreased their feeding rates or the amount of time females spent brooding hatchlings. This may force undernourished nestlings to allocate more energy than is optimal to thermoregulation [14, 35], resulting in slower growth. Nevertheless, alternative causes seem more plausible in our population. In 2010, one year after this study was conducted, we tested if the detrimental effects of human disturbance could be mediated by parental care. We found that fledgling traits decreased as human disturbance increased, but this effect was not related with differences in feeding rate and brooding time (S2 Material). Females may have continued attending nestlings despite disturbance, and the impaired nestling growth could have arisen due to human-induced stress experienced by the offspring. For example, storm petrels (*Hydrobates pelagicus*) breeding near recreational trails suffered increased nestling mortality, although they remain in their holes nests during the periods of human disturbance due to their nocturnal habits [31]. Future research should elucidate the reasons why nesting in cavities may not help birds escape the effects of human disturbance around their nests.

The negative effects of human disturbance on nestling growth were not detected as statistically significant before blue tits reached an age of 11 days. However, this does not mean that the detrimental effects of disturbance started to operate at that age (or at any discrete moment earlier during the nestling period). Our results rather show that all types of nestlings followed growth trajectories that did not abruptly change during the growing period (equal k parameters of all growth curves), but led to different final phenotypes (variable asymptotes associated with disturbance). This supports the idea that the effects detected towards the end of growth are the consequence of disturbances that operated already at the start of growth. Therefore, our results provide further evidence of disturbances faced early in life having detrimental consequences later in life [36–37].

Whatever the mechanisms explaining the impaired development of heavily disturbed blue tits in our study, the fitness consequences are almost certainly important. Unfortunately, monitoring first-year survival is difficult in our population, where fledgling dispersal leads to very low recapture rates (we only recovered one individual in 2010 out of 195 fledged; C. Remacha, pers. obs.). However, in other populations (of blue tits or closely related species) subjected to intensive monitoring, a decrease in fledgling mass of similar magnitude as that observed for heavily disturbed birds has been shown to significantly decrease future survival and fecundity [38–40].

We cannot assure that the critical period of the impact of disturbance after hatching does not extend from the two days considered in our study. For instance, all nestlings from disturbed nests faced at least two periods of stress, because the nestling period exceeds two weeks. Limited sample size in our study prevented us from deeply analysing the ways in which the number of disturbance events impacts on nestlings. However, working-day nestlings from disturbed nests showed similar growth and phenotype as nestlings hatched in quiet nests, and no difference was observed between holiday and working-day nestlings hatched in these control nests. This supports the idea that at least disturbance during the period of early development considered in our study is important. Apart from the evident vulnerability of the hatchling, the parents also increase nest attentiveness with nestling age [41], which may contribute to buffer the impacts of human disturbance on nestling development once the most critical period has been surmounted.

We found various differences between nest types (disturbed or quiet) that were independent of brood type (holiday or working-day). Blue tits breeding near recreation facilities laid their clutches later in the season and suffered lower nestling mortality than those breeding in quiet nests. Reduced nestling mortality in disturbed nests was somewhat unexpected. Nevertheless, variation between types of nest may be due to multiple causes that do not change between brood types. For example, we placed nest boxes in the picnic area the winter before we conducted our study, and newly set nest boxes are known to attract first breeders [42]. However, we did not find evidence that differences in laying dates or breeding performance between types of nests could be attributed to younger or better-quality pairs being attracted to any type of nest box. Other studies that have reported higher breeding success of birds in humanized areas have interpreted such results as a consequence of increased food abundance or reduced predation pressure [43–46]. These effects may have played a role in creating differences between disturbed and quiet nests in our study as well. Disturbed nests are located in picnic areas cleared of undergrowth, which might create variation in food abundance. Predation pressure was also apparently lower in humanized areas in our study: 36.4% of all nests that failed in the woods (quiet nests) were predated, but no nest predation event was recorded in picnic areas (disturbed nests). Although the features that distinguish disturbed and quiet nest locations in our study may be relevant for the distribution and breeding success of blue tits, they do not affect our conclusions regarding the effects of recreation during holidays. The temporal pattern of human influence faced by disturbed nests is the only mechanism that could conceivably create the observed interaction between brood type and nest type.

## Conclusions and Conservation Implications

Our results show that early disturbance events may have negative consequences for wild birds if they overlap with critical stages of development. From the perspective of the offspring, human disturbance can lower individual fitness by decreasing recruitment possibilities of the affected individuals [38–40]. From the perspective of the parents, the increased breeding effort associated with rearing low-quality offspring may not warrant fitness benefits, but still constrains future survival and reproduction [38]. If fitness returns expected from heavily disturbed broods are too small, early abandonment of nests in order to lay a viable replacement clutch in undisturbed habitat might pay off. Importantly, this study reveals an impact of nature recreation that would have remained hidden if the effects of human disturbance had been assessed through nest abandonment alone (which is a typical approach in studies of human impact on breeding birds). The potential significance of these processes should lead us to consider how best to measure human impacts on breeding birds, and highlights the importance of considering fledgling condition when monitoring such impacts in nature recreation areas

From a management perspective, our results suggest that limiting visitor access to natural areas during weekends could help to reduce recreation impacts. Nevertheless, too much restriction may be negatively viewed by the public, and therefore may have unwanted consequences that could translate into less public support for conservation [47–48]. Moreover, recreation facilities like the picnic area we studied may help to keep visitors from uncontrolled dispersal into areas around nature reserves, which might be accompanied by greater impacts [49]. Therefore, other strategies may be more effective, for example setting vegetation barriers [50–51] that may reduce predation risk perceived by birds [52–53], or using knowledge of the response of different species to human disturbance to determine appropriate buffer zones around bird habitat [54]. Based on our study, we can increase our power to reveal human impacts if traditional methods (observation of individual behaviours such as escape decisions, nest abandonment events, etc.) are combined with the monitoring of relevant individual performance parameters.

## Supporting Information

S1 Data Data used in the manuscript. Data of 347 blue tit nestlings monitored until fledging (if they survived) in La Herreria National Heritage Forest during 2009.
(TXT)

S2 Data Data used in the S2 Material. Data of 144 blue tit fledglings in La Herreria National Heritage Forest during 2010 and four nest boxes where nestlings died before age 15 days.
(TXT)

S1 Fig Location of nest boxes in La Herrería National Heritage forest. Circles represent disturbed nests located near recreation facilities in areas designated for picnics. Squares are quiet nest locations in the surrounding woods. The shading of symbols distinguishes between holiday broods (black) and working-day broods (grey). Empty symbols correspond to nest boxes that were not monitored. Except for two (strikethrough) that were initially occupied by blue tits but could not be monitored for different reasons, the rest remained empty or were occupied by other species.
(TIF)

S2 Fig Timing of hatching of all blue tit broods monitored in disturbed (ovals) and quiet nest locations (squares). Filled brood symbols correspond to holiday broods (the calendar bar below brood symbols shows weekends and other public holidays filled in grey). Strikethrough symbols represent failed clutches. Below, line plots show daily variation in temperature (solid line) and rainfall (broken line) in the study area.
(TIF)

S3 Fig Body mass of males and females blue tit fledglings in quiet and disturbed nest locations. Marginal means ± se of body mass for males (grey bars) and females (white bars) in quiet and disturbed nests born on holidays (unhatched bars) and working days (hatched bars). Broken rectangles represent confident intervals for Tukey tests. * *P* < 0.05 *** *P* < 0.001 Significance of the difference compared with the reference level (holiday broods at disturbed nests)
(TIF)

S1 Material Analysis of potential confounding effects. (DOCX)

S2 Material Analysis of human disturbance around nests and nest attentiveness of blue tits during the year 2010. (DOCX)
