# Supplementary figures and images for: Human Disturbance during Early Life Impairs Nestling Growth in Birds Inhabiting a Nature Recreation Area
Source: PLoS One. 2016 Nov 16;11(11):e0166748. doi: 10.1371/journal.pone.0166748 (PMC5112931; doi:10.1371/journal.pone.0166748)

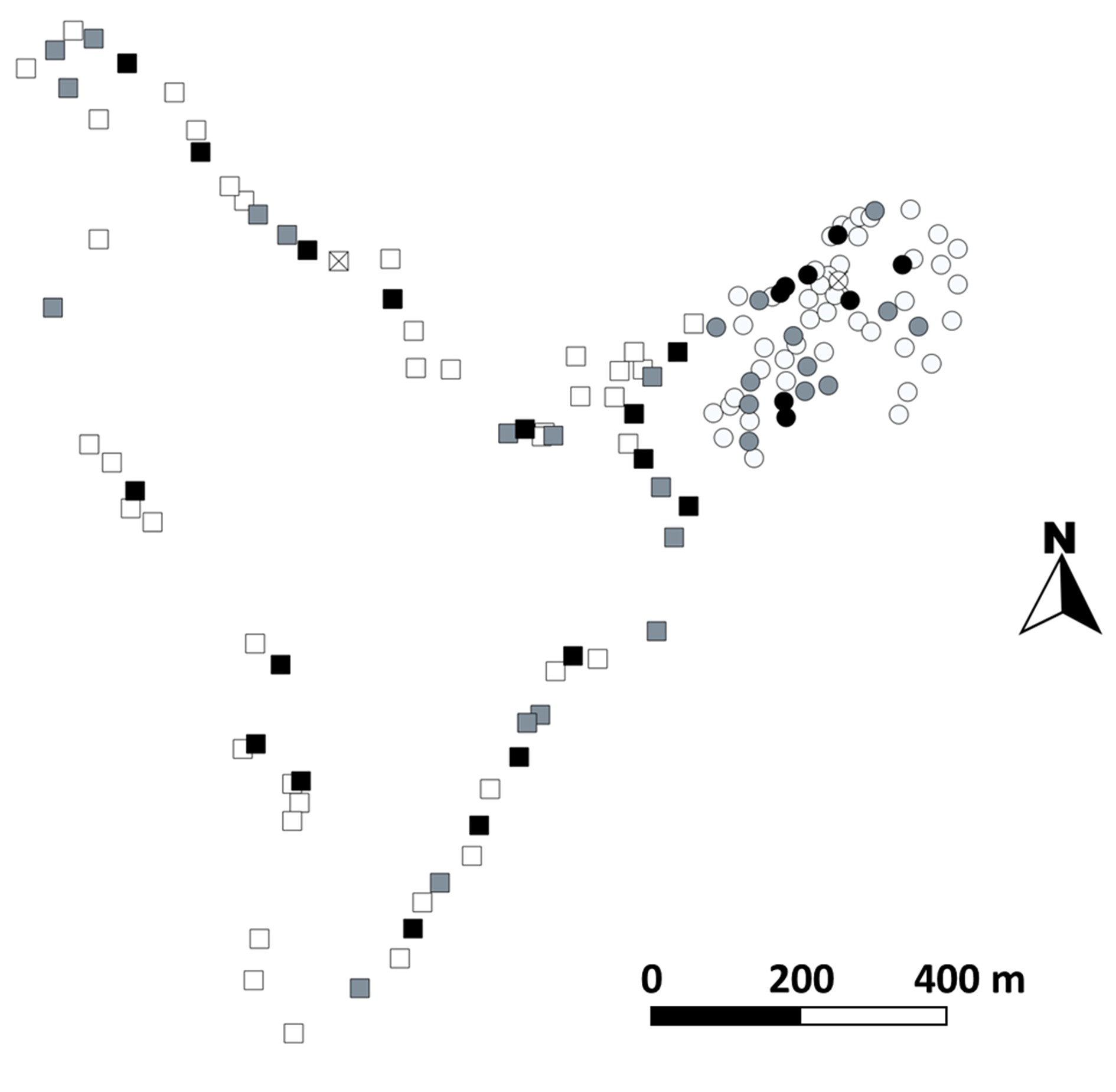

Supplement: S1 Fig — Circles represent disturbed nests located near recreation facilities in areas designated for picnics. Squares are quiet nest locations in the surrounding woods. The shading of symbols distinguishes between holiday broods (black) and working-day broods (grey). Empty symbols correspond to nest boxes that were not monitored. Except for two (strikethrough) that were initially occupied by blue tits but could not be monitored for different reasons, the rest remained empty or were occupied by other species. (TIF) [file pone.0166748.s003.tif]

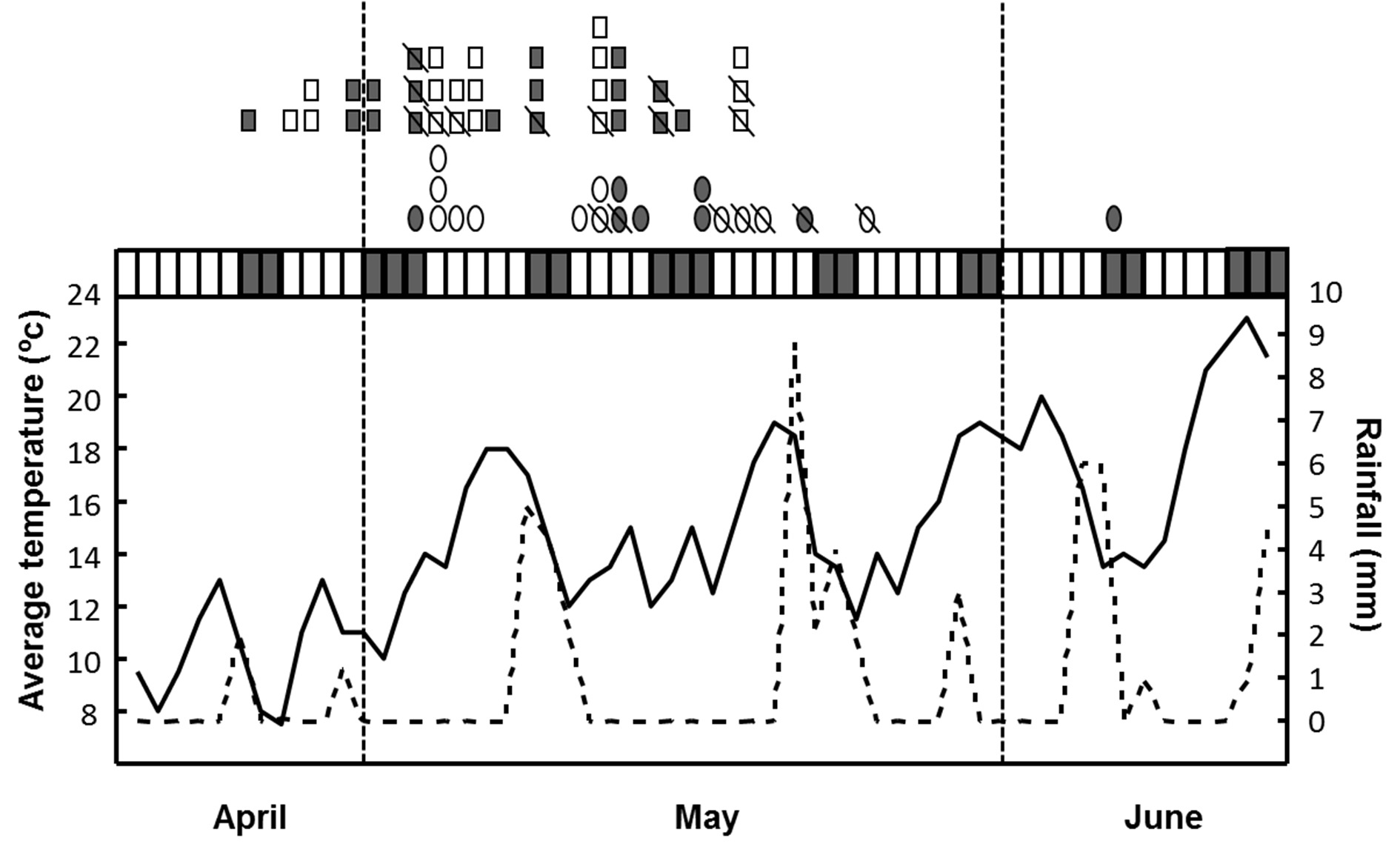

Supplement: S2 Fig — Filled brood symbols correspond to holiday broods (the calendar bar below brood symbols shows weekends and other public holidays filled in grey). Strikethrough symbols represent failed clutches. Below, line plots show daily variation in temperature (solid line) and rainfall (broken line) in the study area. (TIF) [file pone.0166748.s004.tif]

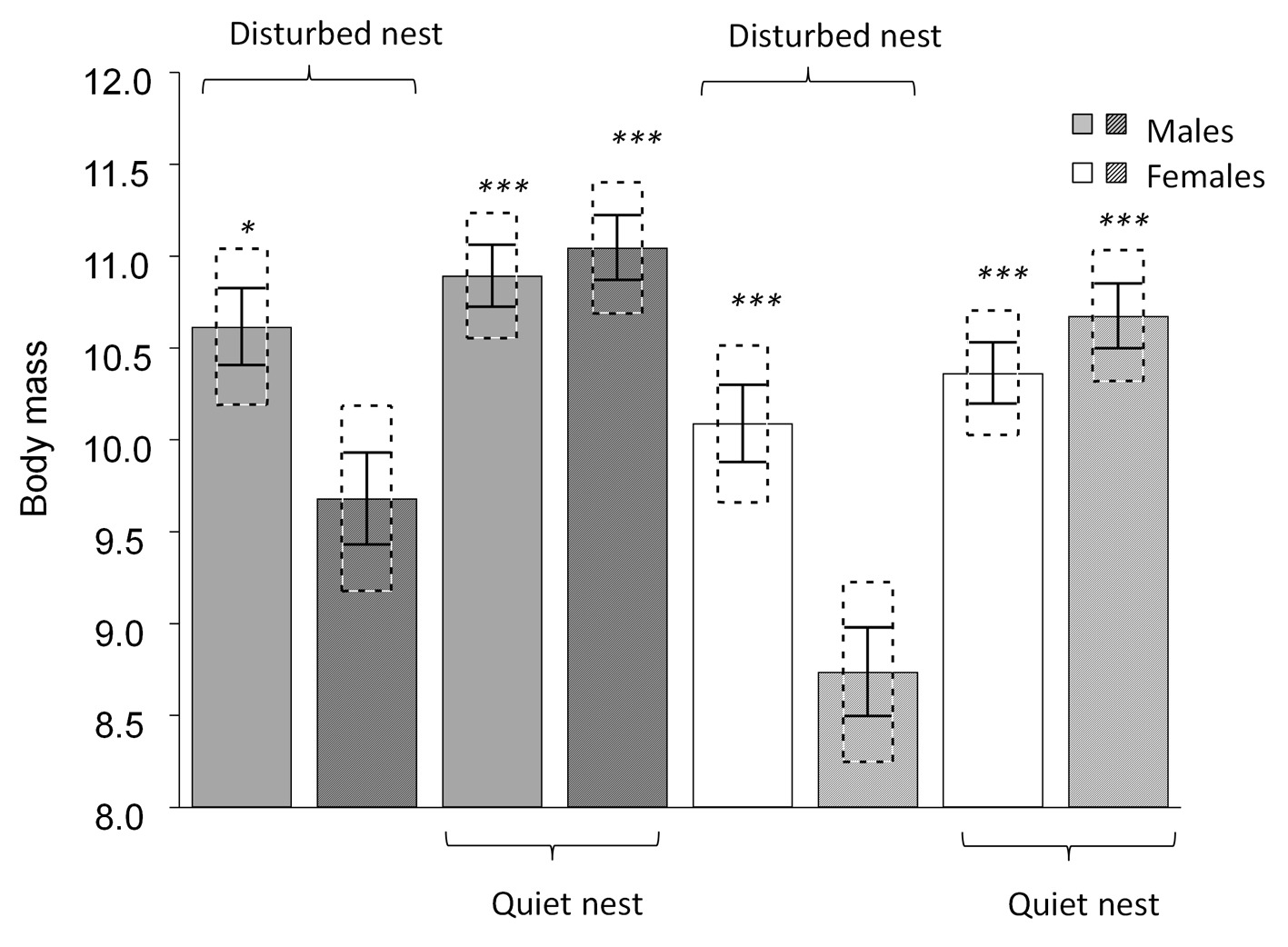

Supplement: S3 Fig — Marginal means ± se of body mass for males (grey bars) and females (white bars) in quiet and disturbed nests born on holidays (unhatched bars) and working days (hatched bars). Broken rectangles represent confident intervals for Tukey tests. * P < 0.05 *** P < 0.001 Significance of the difference compared with the reference level (holiday broods at disturbed nests) (TIF) [file pone.0166748.s005.tif]
