# Supplementary material for: Human Disturbance during Early Life Impairs Nestling Growth in Birds Inhabiting a Nature Recreation Area
Source: PLoS One. 2016 Nov 16;11(11):e0166748. doi: 10.1371/journal.pone.0166748 (PMC5112931; doi:10.1371/journal.pone.0166748)
Supplement: S1 Material — (DOCX) [file pone.0166748.s006.docx]

**S1 Material. Analysis of potential confounding effects.**

Variation between nest types (disturbed or quiet) and brood types (holiday or working-day) in breeding time, weather conditions during hatching, or parental quality of blue tits could potentially confound our results. We analysed these variables to value to what extent they were influential in our study.

*Laying date and weather conditions during hatching*

Broods hatched on average 7 days earlier in quiet than in disturbed nests (F_1,50_ = 11.91; *P* < 0.01; mean ± standard deviation, disturbed: 43.8 ± 8.56, quiet: 37 ± 6.63 days). Average temperature during the two days post-hatching was 1.6 ºC higher for working-day than for holiday broods (F_1,50_ = 7.89; *P* < 0.01; Fig. S1). Nevertheless, interactions between these variables and brood or nest type were not significant in any model (all interactions with *P* > 0.11; Fig. S1), so these differences did not confound our results.

*Parental quality*

We captured the two adults in 34 nest boxes, the female alone in seven cases, and only the male in one case. Of these, 32 females and 29 males bred successfully.

Age of males differed between brood types (χ^2^_(1)_ = 4.47, *P* = 0.03, fathers of holiday broods were more likely to be older breeders), but this difference disappeared when we repeated the test using only successful pairs (those used in the analysis of nestling growth; χ^2^_(1)_ = 2.53, *P* = 0.11). All other effects of nest or brood type and their interactions were not significant for age, body mass or tarsus length of males (all *P* > 0.13). Neither age, body mass or tarsus length of females varied significantly in relation to type of brood or nest box (all effects and interactions had a *P* > 0.09). Clutch size varied between brood types, as holiday clutches were on average one egg larger (8.1 ± 0.29) than working-day clutches (7.2 ± 0.25, F_1,49_ = 5.56, *P* = 0.02), an effect which remained small but statistically significant when it was tested using successful pairs alone (*P* = 0.04).
