# Supplementary material for: Human Disturbance during Early Life Impairs Nestling Growth in Birds Inhabiting a Nature Recreation Area
Source: PLoS One. 2016 Nov 16;11(11):e0166748. doi: 10.1371/journal.pone.0166748 (PMC5112931; doi:10.1371/journal.pone.0166748)
Supplement: S2 Material — (DOCX) [file pone.0166748.s007.docx]

**S2 Material. Analysis of human disturbance around nests and nest attentiveness of blue tits during the year 2010.**

During the study in 2009 of growth trajectories of blue tit nestlings, we did not collect data that could help to identify putative mechanisms to explain how recreational activities may impair nestling development in holiday broods raised in disturbed nests. Possible mechanisms include recreational activities near nest boxes eliciting adult antipredatory behaviours conflicting with parental care [Lima, 2009], or human-induced stress experienced by the offspring compromising nestling growth [Watson *et al.*, 2014]. To improve our understanding of these processes, during 2010 (the year after our study was conducted) we set out to investigate parental decisions of blue tits under variable levels of human disturbance around nest boxes. To this end, we quantified human disturbance around each nest box and measured nest provisioning rates and female brooding as the most relevant parental behaviours during the first days post-hatching in blue tits [Limbourg *et al.*, 2004; García-Navas & Sanz 2011].

We monitored 44 nest boxes occupied by blue tits in 2010. Unfortunately, 15 of these were lost before hatching due to storms occurred during the laying period, and we failed to obtain data of parental care from 6 boxes (due to logistic limitations). These events reduced the sample size to 23 nests, which included all disturbed and most quiet nests that year (S4 Fig). The field methods were the same as in 2009, except for the fact that we sacrificed the monitoring of growth trajectories of nestlings to focus on parental behaviours. Therefore, we only had data of nestling mass at age 15 days.

**S4 Fig**. **Location of nest boxes in La Herrería National Heritage forest in 2010.** Filled circles represent nest boxes occupied by blue tits. Solid black circles represent blue tit nest boxes with data of human disturbance around and nest attendance videos. Solid grey circles represent blue tit nest boxes without data of human disturbance around and nest attendance videos. Open white circles represent unoccupied nest boxes and open white squares represent nest boxes occupied by other species.

We collected data about parental behaviour and human disturbance intensity in each nest box when nestlings were 1 day old (n=5), 2 days old (n=3) or both days (n=15). During an hour, we recorded the behaviour of breeding pairs with a video-camera camouflaged near the nest box (5-10 m). We scored the number of times each adult went into the nest box as the feeding rate and the percentage of time either adult stayed inside the nest box as the brooding time. Both variables characterized nest attendance behaviour. At the same time, an observer located at a safe distance (25-50 m) from the nest box, counted the number of people and vehicles in a radius of 25 m around the nest box, as well as the number of disturbance events (e.g., a group of four people walking near the nest box represented a single disturbance event but also contributed four people to the count of human disturbance load). A Principal Component Analyses (PCA) with these three variables generated a single factor (PC1) that explained 84.3% of variance in the correlation matrix and included all variables with high positive loading (number of disturbance events = 0.97, number of vehicles = 0.85, number of people = 0.93). Therefore, the PC1 was interpreted as a global index of disturbance around nest boxes.

We performed a general linear model with type of nest (disturbed or quiet) and brood (holiday or working-day) as fixed factors and nest-box identity as a random factor. Results showed the expected pattern of variation in disturbance intensity among nest boxes during the two days post-hatching (Fig. S4), nests classified as disturbed faced higher human disturbance levels than those nests classified as quiet. However, disturbed nests differed in realised disturbance levels between holidays (higher disturbance) and working days (lower disturbance). Among quiet nests, the difference between holiday and working day broods was absent, which created a significant nest type × brood type interaction (S5 Fig).


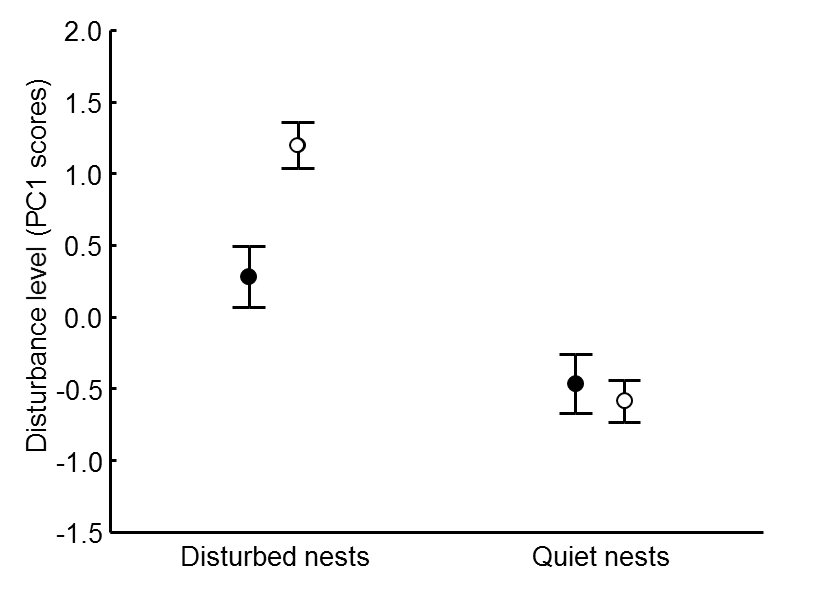


**S5 Fig.** Variation in disturbance levels (PC1 scores, mean ± se) measured around disturbed and quiet nest boxes during the first two days post-hatching of the brood. The different symbols represent holiday (open circles) and working-day broods (filled circles).

Data are available as supporting information (S2 data).

**Statistical procedures**

We performed linear mixed models to test for effects of intensity of human disturbance (PC1) on fledgling body mass, tarsus length and body condition, and general linear models to test for its effect in within-brood variation in fledgling size and mass. In all these models, we used the broods that had been monitored at nestling age 1 day (n = 20), which provided the best balance between homogeneity of the data and statistical power. Nevertheless, we repeated the analyses with the broods measured at nestling age 2 days and found the same results qualitatively. Mixed models were assessed using the package lme4 [Bates *et al.*, 2015]. In all analyses using nestlings as the sample unit, nest identity was included as a random factor. Variables were log-transformed when needed to improve the fit of the models. Non-significant interactions or covariates (laying date, brood size and minimum temperature) were excluded from the models through a stepwise elimination procedure (lmerTest package) [Kuznetsova *et al.*, 2016]. We tested for significance of the effects by log-likelihood ratio test (LRT) comparison between nested models (saturated model versus reduced model). All statistical procedures were performed using R (version 3.3.0).

**Results**

*Breeding success*

Final sample size for the analysis of parental care and intensity of human disturbance was 22 (19 successful and 3 failed broods). For this reason we could not analyse the effect of disturbance on breeding success.

*Fledgling traits*

For body mass, none of the covariates were selected with the stepwise backward elimination process. In the final model, fledgling body mass decreased as human disturbance increased (S6 Fig, S1 Table).

For tarsus length, none of the covariates was selected with the stepwise backward elimination process. In the final model, disturbance effect on tarsus length was close to significance (S1 Table).

Body condition was negatively related with human disturbance (S6 Fig, S1 Table).

**S6 Fig.** Relationship of fledgling body mass (left) and body condition (right) with respect to the level of human disturbance measured around nest boxes (PC1 scores), as estimated in a linear mixed model including nest box as a random effect.

*Sibling inequalities*

The final model to explain within-brood variation in tarsus length included brood size (χ^2^_(1)_ = 4.83; *P* = 0.03) and laying date (χ^2^_(1)_ = 4.13; *P* = 0.04) as covariates. The within-brood CV of body mass (χ^2^_(1)_ = 1.17; *P* = 0.28) and tarsus length (χ^2^_(1)_ = 1.09; *P* = 0.30) did not vary in relation to the level of human disturbance.

*Nest attendance*

The level of human disturbance around nest boxes was not related to feeding rate or brooding time. Nest attendance was only influenced by brood size or minimum temperature (in the case of brooding time; S1 Table).

**S1 Table.** **Reduced model**. Likelihood ratio tests for the effects of level of human disturbance around nest boxes on fledgling traits and parental nest attendance after general mixed models. Nestlings were one or two days old when nest attendance behaviour was measured.

|  |  | χ^2^_(1)_ | *P* |
| --- | --- | --- | --- |
| *Fledgling traits* | |  |  |
| Body mass |  |  |  |
|  | Disturbance level | 12.00 | <0.001 |
| Tarsus length | |  |  |
|  | Disturbance level | 3.52 | 0.06 |
| Body condition | |  |  |
|  | Disturbance level | 7.03 | 0.01 |
| *Nest attendance* | |  |  |
| Feeding rate |  |  |  |
|  | Disturbance level | 1.36 | 0.24 |
|  | Brood size | 14.26 | <0.001 |
| Brooding time |  |  |  |
|  | Disturbance level | 0.04 | 0.84 |
|  | Brood size | 14.55 | <0.001 |
|  | Minimum temperature | 12.58 | <0.001 |

Degrees of freedom = 1 in all cases.

**Conclusions**

We found that fledgling body mass and body condition decreased as human disturbance increased, nevertheless these negative effects on fledgling traits were not related with parental care (feeding and brooding) during the first two days post-hatching. Brood size or minimum temperature were the only variables influencing nest attendance by parents.

**Supplementary references**

Bates D, Maechler M, Bolker B, Walker S. Fitting linear mixed-effects models using lme4. J. Stat. Software. 2015; 67:1-48.

García-Navas V, Sanz JJ. Seasonal decline in provisioning effort and nestling mass of Blue Tits *Cyanistes caeruleus*: experimental support for the parent quality hypothesis. Ibis 2011; 153: 59–69.

Kuznetsova A, Brockhoff PB, Christensen RHB. lmerTest: tests for random and fixed effects for linear mixed effect models (lmer objects of lme4 package). 2016. Available: https://cran.r-project.org/web/packages/lmerTest/index.html

Lima SL. Predators and the breeding bird: behavioral and reproductive flexibility under the risk of predation. Biol. Rev. Camb. Philos. Soc. 2009; 84: 485-513.

Limbourg T, Mateman AC, Andersson S, Lessells CM. Female blue tits adjust parental effort to manipulated male UV attractiveness. Proc. R. Soc. Lond. B 2004; 271: 1903–1908.

Watson H, Bolton M, Monaghan P. Out of sight but not out of harm’s way: Human disturbance reduces reproductive success of a cavity-nesting seabird. Biol. Conserv. 2014; 174: 127-133.
